# In Silico, In Vitro, and In Vivo Assessment of Genotoxicity and Cell Transformation Potential of Gardenia Yellow *E500*

**DOI:** 10.1002/fsn3.72552

**Published:** 2026-11-13

**Authors:** Jiang Liang, Min Qu, Yingying Zhou, Xiaoqiao Tang, Yuan Yuan, Yinjing Zhang, Haibin Xu, Wei Liu, Haitao Han, Lei Zhang, Jintao Li

**Affiliations:** ^1^ NHC Key Laboratory of Food Safety Risk Assessment China National Center for Food Safety Risk Assessment Beijing China; ^2^ Hubei Provincial Key Laboratory for Applied Toxicology Hubei Provincial Center for Disease Control and Prevention Wuhan China; ^3^ Zhanjiang Key Laboratory of Leukemia Pathogenesis and Targeted Therapy Research, Zhanjiang Institute of Clinical Medicine Central People's Hospital of Zhanjiang Zhanjiang Guangdong China; ^4^ Henan Zhongda Hengyuan Biotech Co., Ltd. Luohe China; ^5^ Beijing International Science and Technology Cooperation Base of Antivirus Drug, Beijing Key Laboratory of Environmental and Viral Oncology, College of Chemistry and Life Science Beijing University of Technology Beijing China

**Keywords:** carcinogenicity potential, food colorant, Gardenia Yellow, genotoxicity, safety assessment

## Abstract

Gardenia yellow (GY), a natural food colorant widely used in China, has limited and controversial toxicological evidence, particularly on carcinogenicity and genotoxicity, which hinders its international certification by the Joint FAO/WHO Expert Committee on Food Additives. A high–chromatic–value GY E500 sample was evaluated using an integrated in silico, in vitro, and in vivo strategy following the Integrated Approach to Testing and Assessment framework. Computational predictions of its major components did not identify any consistent evidence for carcinogenicity or genotoxicity. A series of in vitro cell transformation assays, including colony formation, malignant transformation, and soft agar assays, produced no positive findings at 0.1–1 mg/mL, with IC_50_ values of 1.038 and 0.9583 mg/mL in SHEE and HaCaT cells, respectively. Genotoxicity was assessed using the Ames, chromosomal aberration, and micronucleus assays according to Chinese national standards and OECD protocols. No dose‐related increase in revertant colonies was observed, and the micronucleus assay in mice (*n* = 10) showed no significant increase at oral doses up to 6.66 g/kg bw. True structural aberrations did not exceed 5.0% at concentrations up to 5000 μg/mL. Overall, under the conditions of this study, GY E500 showed no evidence of genotoxicity or transformation potential, providing additional evidence for its food additive risk assessment.

## Graphical abstract

An integrated in silico, in vitro, and in vivo assessment of Gardenia Yellow E500 (GY E500), a widely used natural food colorant, revealed no consistent evidence of genotoxicity or cell transformation potential under the tested conditions. Computational predictions, cell transformation assays, and standardized genotoxicity tests yielded concordant findings across multiple lines of evidence. These findings strengthen the toxicological evidence base for GY E500 and support its safety evaluation and risk assessment as a food additive.

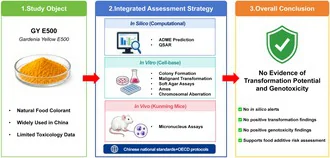

## 1 Introduction

Gardenia yellow (GY) is a fruit extract of *Gardenia jasminoides Ellis*, an evergreen shrub that perhaps originated in China and is on the medicinal and edible plants list of China (Ministry of Health of China 2002; Hong et al. 2015; Xiao et al. 2017; B. Yang and Hui 2013). Due to its notable stability and potent coloring capability, GY is also a natural food colorant widely used in East Asian countries such as mainland China, Japan, and Korea (Shui et al. 2016; Wu et al. 2017). According to the National Standard for the Use of Food Additives (GB 2760–2024) (National Health Commission of the People's Republic of China 2024) in China, GY is approved for use in 22 varieties of food, including fat emulsions, vegetables, biscuits, sweets, coffee, tea and snacks. In recent years, both the production and demand for GY in China have grown steadily. To further facilitate the exportation of GY, some countries including China, are making efforts to obtain certification from the Joint FAO/WHO Expert Committee on Food Additives (JECFA) for international certification as a food additive. Upon JECFA's endorsement of the inclusion of GY in the list of food additives, more comprehensive supporting data were deemed necessary, particularly systematic toxicological data on carcinogenicity and genotoxicity (JECFA 2015).

However, recent studies have presented conflicting findings regarding the safety profile of GY and its components, particularly concerning their potential carcinogenicity and genotoxicity. The main components of GY are 40%–55% (w/w) crocin and 5% alcohol‐soluble impurities, which contain less than 0.5% (w/w) geniposide (Kamikura and Nakazato 1984; Wei et al. 2014). In the Chinese quality specification standard for GY as a food additive, the content of geniposide in GY should not exceed 1% (GB 7912–2010) (Ministry of Health of the People's Republic of China 2010). Evidence from subchronic oral toxicity studies indicates that the liver and kidney are the main target organs of GY toxicity. A 90–day gavage study in Sprague–Dawley rats established a NOAEL of 0.5 g/kg bw for nephrotoxicity (Tang et al. 2020; Wei et al. 2021), and excessive accumulation of geniposide and its metabolite genipin has also been considered a major contributor to hepatotoxicity and nephrotoxicity associated with gardenia–derived products (Yang et al. 2025). By contrast, another subchronic study administering 2% GY orally to mice reported no adverse effects (Li et al. 2017). Studies on gardenia–derived constituents further suggest that geniposide and its metabolite genipin may contribute to hepatic and renal toxicity under certain exposure conditions, indicating that differences in constituent composition may partly account for the inconsistent toxicity findings among GY–related studies (Yang et al. 2025; Tiwari et al. 2025). Genotoxicity findings are likewise inconsistent and appear to vary with the tested material and assay endpoint. Chung et al. reported no overall genotoxicity for GY containing 0.057 mg/g genipin across the Ames, chromosomal aberration, comet, and micronucleus assays, whereas Choi et al. showed that crocin, the major carotenoid constituent of GY, was non–mutagenic and non–clastogenic and reduced H_2_O_2_–induced DNA damage in multiple assay systems (Chung et al. 2009; Choi et al. 2008). In contrast, Ozaki et al. reported negative Ames results but positive rec–assay findings for GY and genipin; GY also increased SCE frequency, with genipin being the only component showing significant SCE induction (Ozaki et al. 2002). These differences highlight the need for a systematic evaluation of GY E500 using complementary genotoxicity and carcinogenesis–related endpoints.

Due to variability in the composition of GY arising from the variety of *Gardenia jasminoides Ellis*, producing areas, and extraction methods, a representative assessment of its genotoxicity and carcinogenicity using samples from the Chinese market is warranted. Modern toxicological assessment has increasingly shifted from reliance on individual tests toward tiered, weight–of–evidence strategies that integrate computational predictions with experimental evidence, with greater emphasis on mechanistic relevance, efficiency, and the 3R principle. Recent studies combining computational modeling with experimental validation in natural bioactive compound research further support the value of integrated evidence in toxicological assessment (Arshad et al. 2025; Sadia et al. 2024, 2026). Accordingly, the OECD Integrated Approach to Testing and Assessment (IATA), together with the increasing use of New Approach Methodologies (NAMs), provides a structured framework for integrating complementary evidence while maintaining regulatory relevance (OECD 2017, 2024a). As an initial evidence layer within this framework, in silico predictive toxicology, propelled by the TOX21 program (Thomas et al. 2018), relies heavily on QSAR modeling, which is widely used to predict absorption, distribution, metabolism, excretion, and toxicity (ADMET) properties (Han et al. 2019; Kiran et al. 2025), as well as carcinogenicity and genotoxicity, when experimental data are limited (Battistelli and Bossa 2025; OECD 2024b). Colony–forming unit assays, malignant cell transformation tests, and soft agar colony formation assays have been recognized by the European Union as validated tools for carcinogenicity evaluation and are widely applied in integrated carcinogenic hazard assessment (EURL ECVAM 2011).

In the present study, an integrated, stepwise strategy combining in silico, in vitro and in vivo approaches was employed to assess the genotoxicity and cell transformation potential of GY E500 using a representative high–chromatic–value sample from the Chinese market. This strategy was developed based on the tiered IATA framework and Chinese national standards aligned with OECD genotoxicity testing guidelines. Computational prediction was used to characterize the ADMET properties and potential toxicity alerts of the major components of GY, while cytological transformation assays and standardized genotoxicity tests were conducted to evaluate carcinogenesis cellular responses and genotoxicity. By integrating complementary evidence across computational and experimental levels, this strategy reduces reliance on individual assays and supports a more coherent weight–of–evidence assessment of GY E500. This study provides additional safety evidence for the food additive risk assessment of GY E500, thereby supporting its evaluation under the JECFA framework and its potential international certification as a food additive.

## 2 Materials and Methods

### 2.1 Materials and Chemicals

The GY E500 sample (Lot No. 20150529, chromatic valence 510) was obtained from Henan Zhongda Hengyuan Biotech Co. Ltd. (Henan, China). Food‐grade ethanol (80% v/v, Lot No. 20141223) was purchased from Henan Xinheyang Alcohol Co. Ltd. (Henan, China). Sodium carboxymethyl cellulose was obtained from Sinopharm Chemical Reagent Co. Ltd. (Wuhan, China).

Cell lines used in this study included SHEE cells (human esophageal epithelial immortalized cell line, provided by the Institute for Viral Disease Control and Prevention, Chinese CDC) and HaCaT cells (human keratinocyte line, purchased from the Institute of Basic Medical Sciences, Chinese Academy of Medical Sciences, Beijing, China). Epithelial–derived cell models were used because epithelial cells are among the first cellular barriers exposed to substances after oral intake and may reflect early cellular responses. CHO–K1 cells (Chinese hamster ovary cell line) were obtained from the China Typical Culture Preservation Center (CCTCC GDC0018, Wuhan University, China). All cells used in the in vitro assays were within 30 passages.

Cell culture reagents DMEM/F12 medium, fetal bovine serum, and penicillin–streptomycin (P.S.), all purchased from Hyclone (Carlsbad, CA, USA). The Cell Counting Kit–8 (CCK‐8) was purchased from Dojindo Laboratories (Kumamoto, Japan).

Chemicals and reagents for genotoxicity assays included sodium azide and 2–aminofluorene (Amresco and Sigma–Aldrich, USA), Dexon and 1,8–dihydroxyanthraquinone (Dr. Ehrenstorfer GmbH, Germany), cyclophosphamide (Baxter Oncology GmbH, Germany), ethyl methanesulfonate, diethylnitrosamine (DEN), and Giemsa stain (Sigma–Aldrich, USA). The S9 metabolic activation system from Aroclor 1254–induced male Sprague–Dawley rats was obtained from MOLTOX Molecular Toxicology Inc. (Boone, NC, USA).

### 2.2 Preparation and Characterization of the Test Substance

GY E500 sample (Lot No. 20150529) with a chromatic valence of 510 was provided. The dried ripe fruit of *Gardenia jasminoides* harvested in Hubei province was ground into fine pieces and extracted with 80% (V/V food grade alcohol, Lot No. 20141223). The extract was concentrated using macroporous resin and spray–dried at an inlet air temperature of 180°C–210°C and an outlet air temperature of 70°C–110°C to obtain the final colorant powder. The final product of GY E500 was an orange–red powder, extracted from dried fruits in a proportion of 1:50. No toxic chemicals were involved during production. The crocetin and its derivatives, as well as other derivative components of GY E500 were determined by HPLC–ACPI–MS/MS method, which has been patented in both China (Patent No. 201610124837.6) and the US (US Patent 2017/0248561 A1) (Hui et al. 2017). HPLC–MS/MS analysis was performed on a C18 column (250 × 4.6 mm, 5 μm) with a water–acetonitrile–acetic acid/acetonitrile gradient at a flow rate of 0.8 mL/min, and detection was set at 440 nm. Mass spectrometric conditions were: spray voltage −4 kV, sheath gas 30 arb, auxiliary gas 10 arb, sweep gas 0 arb, capillary temperature 350°C, operated in negative ion mode with collision–induced dissociation (CID) energy 35%, and scan range m/z 150–2000. As shown in Figure 1B, the components of GY E500 sample were analyzed by HPLC chromatogram. The composition of GY E500 was characterized as follows: the content of crocin, crocetin and their derivatives was 44.82% (w/w), while the content of geniposide and genipin was 0.214% and 0.03% (w/w), respectively. The average ash and moisture contents were 1.85% and 2.78%, respectively (Tang et al. 2020). GY E500 was prepared using ultrapure water as the stock solution. The stock solution was diluted with DMEM/F12 medium to achieve the desired concentrations for different in vitro experiments.

**FIGURE 1:**
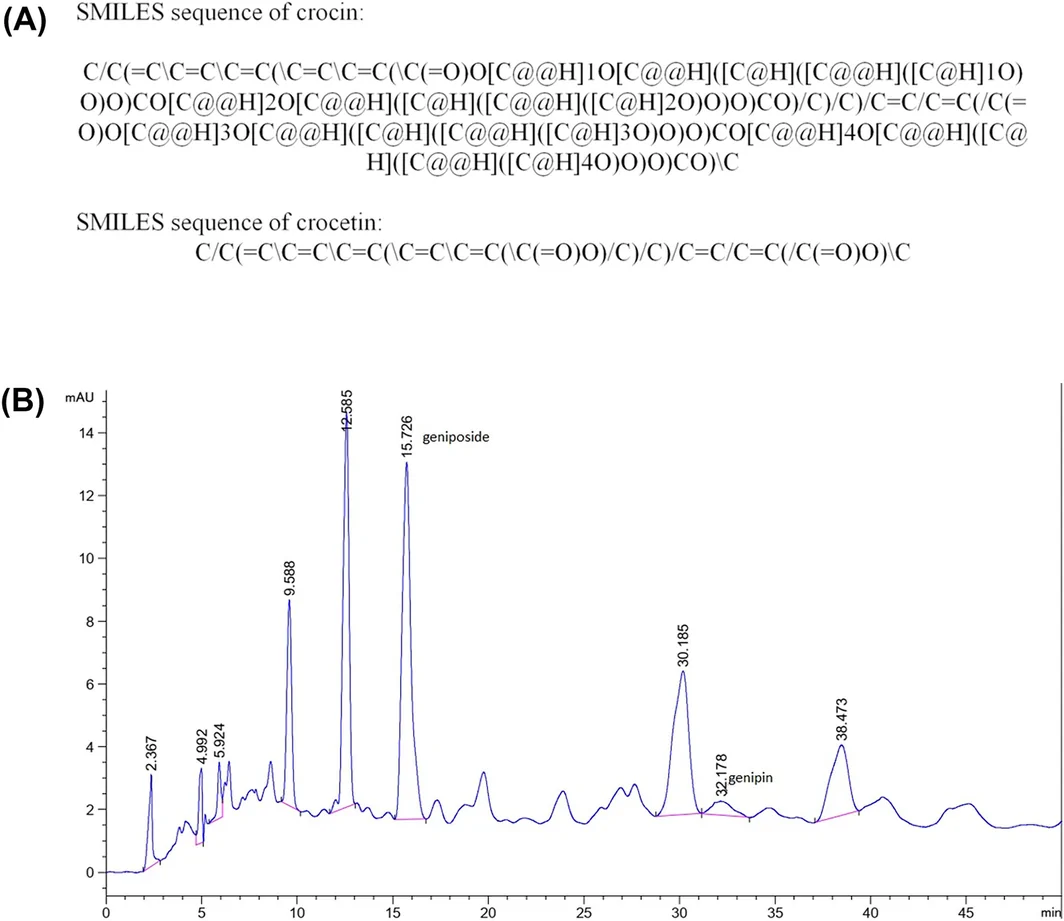
(A) SMILES sequences of crocin and crocetin, two major constituents of gardenia yellow. (B) HPLC chromatogram of the GY E500 sample, showing the separation and identification of representative compounds. Peaks corresponding to geniposide and genipin are indicated, along with retention times.

### 2.3 In Silico Toxicological Prediction

In order to obtain comprehensive ADMET information on the identified compounds of GY, we used two publicly available QSAR platforms, ADMETlab 2.0 (https://admetmesh.scbdd.com) and admetSAR 2.0 (http://lmmd.ecust.edu.cn/admetsar2/). Toxicity prediction was performed using the SMILES strings of crocin and crocetin obtained from PubChem and consistently applied across both platforms (Figure 1A), and results from both platforms were compared to guide subsequent experiments. ADMETlab 2.0 is a peer–reviewed resource that aggregates large, curated experimental datasets from major bioactivity databases and implements validated QSAR models across ADMET endpoints (Xiong et al. 2021). admetSAR 2.0 is a peer–reviewed QSAR resource that compiles a large ADMET knowledgebase and employs machine–learning models with documented external validation for multiple endpoints (Cheng et al. 2012; Yang et al. 2019). Further details about both platforms are provided in the Supplementary Methods.

### 2.4 Cell Transformation Potential Evaluation

#### 2.4.1 Cytotoxicity Test

The cytotoxic effects of GY E500 were assessed using the Cell Counting Kit–8 (CCK‐8). SHEE cells and HaCaT cells were used to evaluate the cytotoxicity of GY E500.

Cells were seeded into 96–well plates at a density of 2000 cells/well and allowed to adhere overnight. They were then treated with graded concentrations of GY E500 (0, 0.1, 0.5, 1, 2, 4, 6, 8, and 10 mg/mL) for 24 h. After incubation, 10 μL of CCK–8 solution was added to each well and incubated for 2 h at 37°C, followed by absorbance measurement at 450 nm. Cell viability was calculated relative to the untreated control. The experiment was independently repeated three times.

The inhibition rate (%) of cell viability was calculated using the following Equation (1):

(1) $$\text{Inhibition rate}\;\left(\%\right)=\frac{A_{\text{control}}-A_{\mathrm{GY}\;E500}}{A_{\text{control}}-A_{\text{blank}}}\times 100\%$$

where $\;A_{\text{control}}$ is the absorbance of the untreated control group, $A_{\mathrm{GY}\;E500}$ is the absorbance of the GY E500–treated group at different concentrations, and $A_{\text{blank}}$ is the absorbance of the blank wells without cells.

The CCK‐8 assay was used to characterize cytotoxicity and guide concentration selection for subsequent cell transformation potential assays.

#### 2.4.2 Colony–Forming Unit Assays

The colony–forming unit assay, SHEE and HaCaT cells in the logarithmic growth phase were seeded into 6–well plates at a density of 400 cells/well and cultured in a 5% CO_2_ incubator at 37°C. After 24 h, different concentrations of GY E500 solution (0, 0.1, 0.5, 1.0 mg/mL) were treated at 2 mL/well. The cells were then cultured for 7 days, with the medium being replaced on day 4. On day 7, the cells were washed twice with phosphate–buffered saline (PBS), fixed with methanol for 20 min, and stained with Giemsa solution. The experiment was independently repeated three times. Colonies containing more than 50 cells were counted under an inverted optical microscope, and the colony formation efficiency was calculated according to Equation (2), as described in established clonogenic assay protocols (Franken et al. 2006):

(2) $$\text{Colony formation efficiency}\left(\mathrm{CE}\right)=\frac{N_{\text{colonies}}}{400}\times 100\%$$

where $N_{\text{colonies}}$ is the number of counted colonies per well.

#### 2.4.3 Malignant Transformation

The SHEE and HaCaT cells in the logarithmic growth phase were inoculated into a T25 culture flask, 1 × 10^4^ cells per flask, and cultured with DMEM medium supplemented with 1% P.S. and 10% FBS in a 5% CO_2_ incubator at 37°C for 24 h. DEN–treated (100 μmol/L) SHEE cells for 7 days were included as the positive control under identical conditions.

Subsequently, GY E500 solution of different concentrations was added to each flask, with the solution being replaced on day 4. On days 7, 10, and 14, the medium was replaced with DMEM/F12 medium containing 0.1 μg/mL TPA (12–O–Tetradecanoylphorbol–13–acetate), supplemented with 2% FBS, 0.5% P.S., 0.5% Glu, and 0.2% ITEs. The cells were then continued cultured in this medium, with the medium changed every three days until week 4. A vehicle–only negative control (0 mg/mL GY E500) was included in parallel and was subjected to the same medium–change regimen and TPA (0.1 μg/mL) schedule as the treatment groups. The cells were fixed with methanol and stained with Giemsa. The experiment was independently repeated three times. The number of transformed *foci* was counted using an inverted optical microscope (OECD 2007; EURL ECVAM 2011), and the cell transformation frequency (TF) was calculated according to Equation (3):

(3) $$\mathrm{TF}\;\left(\times 10^{-4}\right)=\frac{\text{Average number of transformation foci}\;\mathrm{per}\;\text{flask}}{\mathrm{CE}\times 2}$$

where CE denotes the colony formation efficiency from Equation (2). The “×2” factor corrects for the twofold dilution introduced by mixing the cell suspension with the staining solution at a 1:1 ratio.

#### 2.4.4 Soft Agar Colony Formation Experiment

Following malignant transformation, cells were further assessed for anchorage–independent growth using a soft agar colony formation assay (Borowicz et al. 2014). To prepare the agarose base layer, 1 mL of double–concentrated DMEM culture medium (20% FBS, 2% P.S.) was mixed with 1 mL of 1.2% agarose (40°C). After mixing, the solution was transferred into a 6–well cell culture plate for solidification at room temperature and subsequently incubated in a 5% CO_2_ incubator at 37°C.

To prepare the agarose inoculation layer, 0.5 mL of double–concentrated DMEM culture medium (20% FBS, 2% P.S.) was mixed with 0.5 mL of 0.7% agarose, followed by the addition of 1 × 10^3^ cells/0.2 mL. The mixture was added onto the solidified base layer to form a double–layer agar system and incubated at 37°C in a 5% CO_2_ incubator for 10 days. Colony formation was examined under an inverted light microscope. The experiment was independently repeated three times.

### 2.5 Genotoxicity Assessment

#### 2.5.1 Bacterial Reverse Mutation Assay (Ames Test)

Histidine mutant *Salmonella typhimurium* strains TA97a and TA98 (to detect frame–shift mutagens) and strains TA100, TA102 and TA1535 (to detect base–pair substitution mutagens) were used as tester strains. All strains were obtained from MOLTOX Molecular Toxicology Inc. (North Carolina, USA) and passed the required strain–characterization checks before use. All solutions were sterilized by autoclave or microporous filtration membrane with a diameter of 0.22 μm.

The mutation assay was performed using the plate–incorporation method according to the National standards for food safety of the Republic of China: Bacterial Reverse Mutation Test (GB 15193.4–2014) (National Health and Family Planning Commission of the People's Republic of China [NHFPC] 2014a), which was developed based on OECD Test Guideline 471 (OECD 2020). The positive controls used in the Ames assay were sodium azide, dexon, 2–aminofluorene, and 1,8–dihydroxyanthraquinone. A 0.1 mL aliquot of GY E500 at 128, 320, 800, 2000, 5000 μg per plate, 0.5 mL of S9 mix (or ultrapure water, for the S9 negative group), and 0.1 mL inoculum of the tester strain were added to each tube containing 2 mL of top agar. The mixtures were poured onto the minimal agar plates and incubated at 37°C for 52 ± 4 h. Triplicate plates were run for each assay.

#### 2.5.2 In Vitro Chromosomal Aberration Assay

Cyclophosphamide (CP) and ethyl methanesulfonate (EMS) were used as positive controls. In vitro chromosomal aberration assay was performed using CHO–K1 cells, following the method described in the National Standard for Food Safety of the People's Republic of China (GB 15193.23–2014) (NHFPC 2014b), which was developed based on OECD Test Guideline 473: In Vitro Mammalian Chromosomal Aberration Test (OECD 2016a). Cells were divided into 5 groups including a negative group (DMEM/F12 with or without S9), a positive group (CP with S9 and EMS without S9), and 3 dosage groups, i.e., 5.00 mg/mL, 2.50 mg/mL, and 1.25 mg/mL (with or without S9). After 48 h of culture, the cells were incubated with the corresponding control or GY E500–containing serum–free medium for 4 h in the presence or absence of S9. Then, the cells were washed three times with PBS and subsequently cultured in complete medium containing 10% fetal bovine serum. Approximately 20 h post–treatment initiation, colcemid solution was administrated to each culture at a final concentration of 1 μg/mL for 4 h. Cells were subjected to potassium chloride hypotonic treatment, fixed with Carnoy fixative, and stained with Giemsa for metaphase analysis. Two hundred metaphases were selected and examined for each treatment group under 1000× magnification using a light microscope. The results were expressed as the mean aberrant metaphases excluding gaps and multiploids per 100 metaphases.

#### 2.5.3 In Vivo Micronucleus Test

The in vivo micronucleus test was conducted according to the National Standard for Food Safety of the People's Republic of China (GB 15193.5‐2014) (NHFPC 2014c), which was developed based on OECD Test Guideline 474: Mammalian Erythrocyte Micronucleus Test (OECD 2016b).

Specific pathogen–free Kunming mice, aged 7–12 weeks, weighing between 25 and 30 g, of both sexes were used. The study complied with national regulations on laboratory animal welfare and was approved by the Institutional Animal Use and Management Committee (Approval No. YZC–201601004). Five same–sex animals were housed per individually ventilated cage under controlled conditions, including a 10 h light/14 h dark cycle, a temperature of 23°C ± 2°C, relative humidity of 55% ± 15%, and ad libitum access to standard rodent diet and tap water. After 3 days of acclimatization, the animals were randomly assigned to five groups, each containing five males and five females, using a random number table to balance sex and body weight.

GY E500 powders were dissolved in 0.5% sodium carboxymethyl cellulose solution to prepare the dosing solutions. The negative control group received 0.5% sodium carboxymethyl cellulose solution, and cyclophosphamide at 0.04 g/kg bw was used as the positive control. The daily oral dosages of GY E500 were 1.665, 3.330, and 6.660 g/kg bw, corresponding to 1/4, 1/2, and the maximum feasible dose, respectively. GY E500 and the vehicle control were administered once daily for two consecutive days with a 24 h interval. The highest dose of 6.66 g/kg bw was determined based on the maximum solubility of GY E500 (33.3%) and the maximum gavage volume (20 mL/kg bw), supported by previous acute oral toxicity data showing an LD50 greater than 15.0 g/kg bw (Tang et al. 2020). Mice were euthanized by cervical dislocation 6 h after the final administration, and the femurs were collected for bone marrow preparation. At least two bone marrow smears per animal were prepared. The air–dried slides were fixed in methanol for 5–10 min and subsequently stained with Giemsa for 10–15 min. The slides were then examined under 1000× magnification. Micronuclei were identified as small round or oval bodies, ranging from 1/5 to 1/20 of the diameter of polychromatic erythrocytes (PCE). A minimum of 200 erythrocytes, including both PCEs and normochromatic erythrocytes (NCEs), were examined per animal to calculate the PCE/(PCE + NCE) ratio according to Equation (4). A total of 2000 PCEs per animal were scored by the same observer to determine the frequency of micronucleated polychromatic erythrocytes (MNPCEs), calculated according to Equation (5).

(4) $$\mathrm{PCE}/\left(\mathrm{PCE}+\mathrm{NCE}\right)\;\text{ratio}\;\left(\%\right)=\frac{N_{\mathrm{PCE}}}{N_{\mathrm{PCE}}+N_{\mathrm{NCE}}}\times 100\%$$

(5) $$\text{MNPCE ratio}\;\left(‰\right)=\frac{N_{\text{MNPCE}}}{N_{\mathrm{PCE}}}\times 1000$$

### 2.6 Statistical Analysis

The statistical analyses were conducted using SPSS 22.0. Continuous data were expressed as mean ± SD. Normality was assessed using the Shapiro–Wilk test, and homogeneity of variance was assessed using Levene's test before parametric analyses. Continuous data, such as cell viability and transformation frequency (TF), were analyzed using one–way ANOVA. Dunnett's post hoc test was used to compare each treatment group with the corresponding control group. IC_50_ values were derived from four–parameter logistic fitting of cytotoxic dose–response curves, and concentration–related TF trends were assessed using the Jonckheere–Terpstra test. For pairwise comparisons between two groups, Student's *t*–test was applied. Frequency data, such as chromosomal aberrations and micronucleus frequencies, were analyzed using the chi–square test. The statistically significant level (*p*–value) was set at 0.05.

## 3 Results

### 3.1 In Silico Toxicity Predictions of GY

#### 3.1.1 Animal Toxicity Predicton of Crocin and Crocetin

The predictions of animal toxicity for crocin and crocetin, the main components of GY, are presented in Table 1. According to the findings from ADMETlab 2.0, crocin has the potential to induce certain hepatic damage and skin allergies; however, it does not exhibit any toxicity in genotoxicity. Consistent with these results, data from admetSAR 2.0 suggest that crocin may exhibit certain hepatotoxic and cardiotoxic properties. Nonetheless, it is neither carcinogenic nor genotoxic, and it does not induce any corrosive or irritative effects on the eyes. Similarly, the findings from ADMETlab2.0 suggested that crocetin exhibits certain hepatotoxicity, potentially resulting in liver damage, and is also associated with skin allergies as well as a certain level of eye irritation. Additionally, there is a slight possibility of respiratory toxicity. Crocetin also does not exhibit genotoxic or carcinogenic potential. In alignment with these observations, data from admetSAR2.0 reveal that crocetin, from saffron acid, may not only cause certain hepatotoxicity but also may induce corrosion and irritation to the ocular surfaces. Nevertheless, no evidence of carcinogenicity or genotoxicity was observed.

**TABLE 1:** ADMETlab2.0 and admetSAR2.0 predicted crocin and crocetin animal toxicity results.

Platform	ADMETlab2.0			admetSAR2.0		
Component	Property	Result^a^	Possibility^b^	Property	Result	Possibility
Crocin	hERG blockers	−−	0.101	Carcinogenicity	−	0.9571
	H‐HT (Human hepatotoxicity)	−−	0.309	Eye corrosion	−	0.9884
	DILI (Drug induced liver injury)	++	0.84	Eye irritation	−	0.9012
	AMES toxicity	−−	0.122	Ames mutagenesis	−	0.54
	Rat oral acute toxicity	−−−	0.03			
	FDAMDD (Maximum recommended daily dose)	−−−	0.003	hERG Inhibitor (cardiotoxicity)	+	0.7367
	Skin sensitization	+++	0.909			
	Carcinogencity	−−−	0.047	Micronucleus test	−	0.6641
	Eye corrosion	−−−	0.003			
	Eye irritation	−−−	0.022	Hepatotoxicity	+	0.525
	Respiratory toxicity	−−−	0.014			
Crocetin	hERG Blockers	−−−	0.05	Carcinogenicity	−	0.6571
	H‐HT (Human hepatotoxicity)	++	0.815			
	DILI (Drug induced liver injury)	+++	0.913	Eye corrosion	+	0.6217
	Ames toxicity	−−−	0.007			
	Rat oral acute toxicity	−	0.377	Eye irritation	+	0.7379
	FDAMDD (Maximum recommended daily dose)	−−−	0.026	AMES mutagenesis	−	0.56
	Skin sensitization	++	0.74	hERG Inhibitor (cardiotoxicity)	−	0.5
	Carcinogencity	−	0.406			
	Eye corrosion	−−−	0.009	Micronucleus test	−	0.8
	Eye irritation	++	0.837			
	Respiratory toxicity	+	0.549	Hepatotoxicity	+	0.55

^a^ “+” indicates positive/toxic, “−” indicates negative/non‐toxic.

^b^ Probability refers to the probability of the result, which can be considered as the confidence level of the result.

#### 3.1.2 QSAR Structure–Activity Relationship Prediction of Crocin and Crocetin

The predictions of the structure–activity relationship for crocin and crocetin, based on the specific assessments of the ADMETlab 2.0 platform, are shown in Table 2. For crocetin, the results indicate one structure with potential carcinogenic or genotoxic potential, and three structures for skin sensitizing potential. In the case of crocetin, the evaluation of crocetin identified one structure for skin‐sensitization and two structures deemed toxic for aquatics; however, no alert structures for carcinogenicity or genotoxicity were observed.

**TABLE 2:** ADMETlab 2.0 predicted crocin and crocetin toxic group results.

Component	Property	Value	Comment
Crocin	Acute toxicity rule	0 alerts	20 substructures; acute toxicity during oral administration
	Genotoxic carcinogenicity rule	1 alerts	117 substructures; carcinogenicity or mutagenicity
	Non genotoxic carcinogenicity rule	0 alerts	23 substructures; carcinogenicity through nongenotoxic mechanisms
	Skin sensitization rule	3 alerts	155 substructures; skin irritation
	Aquatic toxicity rule	5 alerts	99 substructures; toxicity to liquid (water)
	Non biodegradable rule	1 alerts	19 substructures; non‐biodegradable
	SureChEMBL rule	2 alerts	164 substructures; MedChem unfriendly status
Crocetin	Acute toxicity rule	0 alerts	20 substructures; acute toxicity during oral administration
	Genotoxic carcinogenicity rule	0 alerts	117 substructures; carcinogenicity or mutagenicity
	Non Genotoxic carcinogenicity rule	0 alerts	23 substructures; carcinogenicity through nongenotoxic mechanisms
	Skin sensitization rule	1 alerts	155 substructures; skin irritation
	Aquatic toxicity rule	2 alerts	99 substructures; toxicity to liquid(water)
	NonBiodegradable rule	0 alerts	19 substructures; non‐biodegradable
	SureChEMBL rule	2 alerts	164 substructures; MedChem unfriendly status

### 3.2 In Vitro Cell Transformation Potential of GY E500

#### 3.2.1 Cytotoxicity Test

As illustrated in Figure 2, the inhibitory effect of GY E500 on SHEE and HaCaT cells demonstrated a clear dose–dependent enhancement, with the inhibition rate increasing along with the concentration of GY E500. The half–inhibitory concentrations (IC_50_) of GY E500 for SHEE and HaCaT cells were 1.038 mg/mL and 0.959 mg/mL, respectively. These results indicate that SHEE and HaCaT cells exhibited comparable cytotoxic sensitivity to GY E500, supporting the rational use of both models for subsequent transformation experiments. Accordingly, concentrations of 0.1, 0.5, and 1 mg/mL were selected for the cell transformation assays.

**FIGURE 2:**
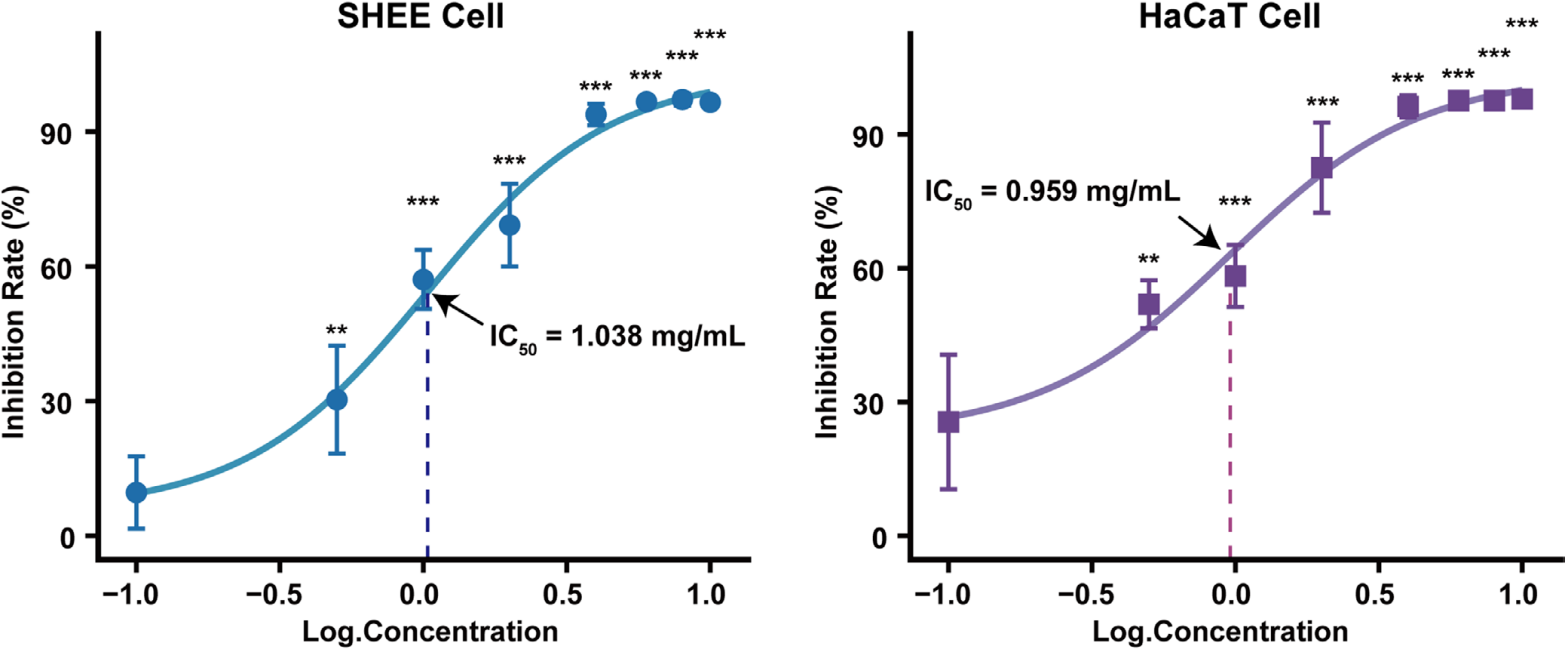
Effects of GY E500 on cell viability in SHEE and HaCaT cells after 24 h of exposure (0.1–10 mg/mL). Data represent mean ± SD (*n* = 3). **p < 0.01 and ***p < 0.001, compared with the control group.

#### 3.2.2 Colony–Forming Uunit Assays and Malignant Transformation

As shown in Figure 3 and Table S1, the TF of SHEE and HaCaT cells was evaluated after exposure to GY E500 within the non–cytotoxic concentration range. Compared with the negative control, GY E500–treated groups showed increased TF values in both cell models, with statistically significant differences observed based on one–way ANOVA followed by Dunnett's test. Among the GY E500–treated groups, the Jonckheere–Terpstra test showed a significant increasing trend in HaCaT cells (*p* = 0.0006), but not in SHEE cells (*p* = 0.363). These results indicate that although GY E500 treatment was associated with elevated TF values, the concentration–related response was not consistently observed across both cell models. Figure 4A shows the morphological differences between SHEE cells and positive control cells in the malignant transformation assay, revealing significant morphological differences between them.

**FIGURE 3:**
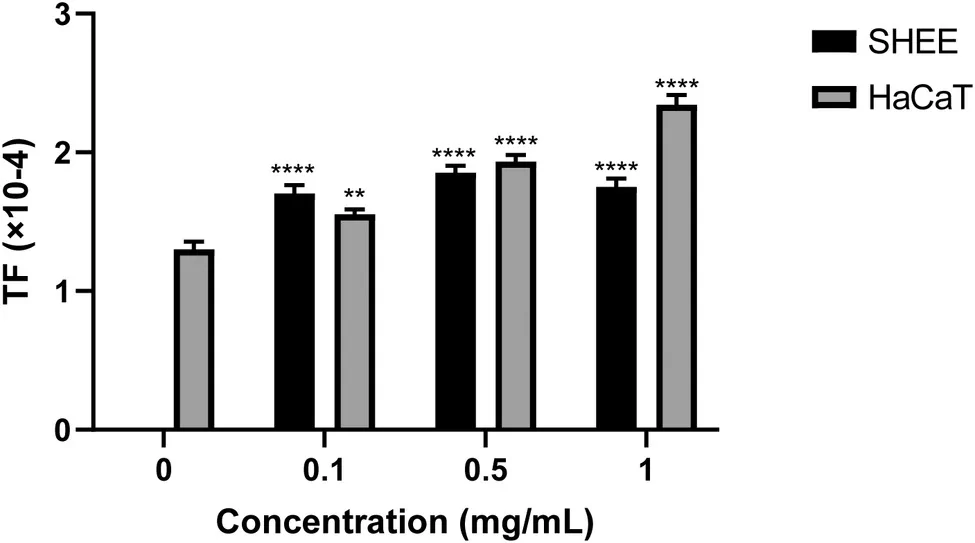
Malignant transformation frequency (TF, ×10^−4^) in SHEE and HaCaT cells following exposure to GY E500 (0, 0.1, 0.5 and 1 mg/mL). Data represent mean ± SD (*n* = 3). Statistical significance was evaluated by one‐way ANOVA; **p < 0.01 and ****p < 0.0001, compared with the control group.

**FIGURE 4:**
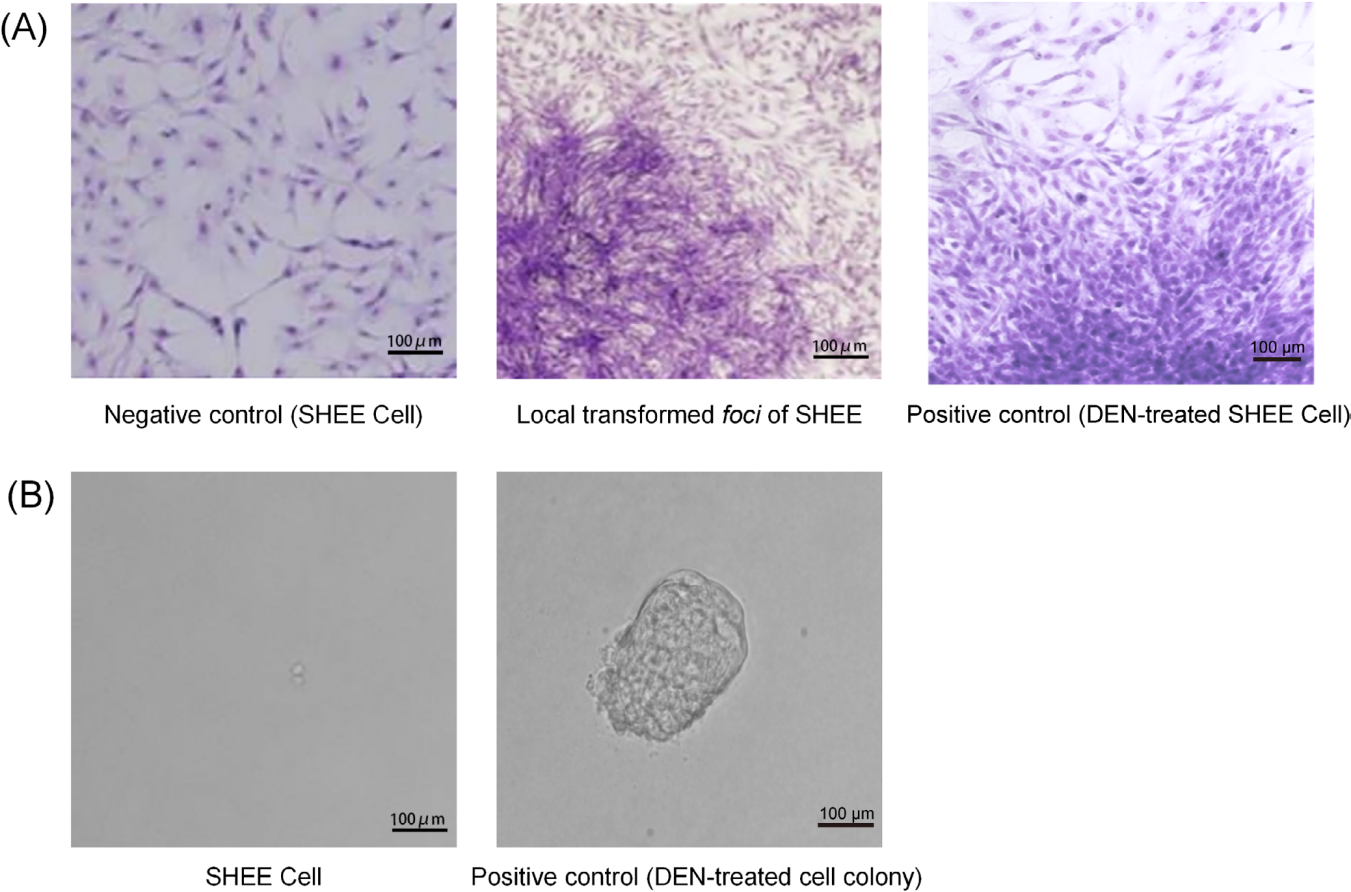
Representative images of assays evaluating the cell transformation potential of GY E500. (A) Malignant transformation assay in SHEE cells. Left: Negative control (untreated SHEE cells); right: Local transformed *foci* in SHEE cells after exposure to GY E500. (B) Soft agar colony formation assay in SHEE cells. Left: Negative control (untreated SHEE cell); right: Positive control (DEN‐treated SHEE cell colony). All experiments were independently repeated three times. Scale bar = 100 μm.

#### 3.2.3 Soft Agar Colony Formation Experiment

As shown in Figure 4B, SHEE cells and HaCaT cells after different treatments were subjected to the soft agar colony formation assay. Colony formation was observed under a microscope after 10 days of cultivation. The results indicated that, except for DEN–treated SHEE cells, which exhibited distinct colony formation, control cells and 0.5 mg/mL GY E500–treated SHEE and HaCaT cells failed to form colonies in the soft agar medium.

The focus–formation assay evaluates morphological transformation, whereas the soft agar assay assesses anchorage–independent growth, a functional characteristic associated with malignant transformation. Although GY E500 significantly increased TF, the concentration–related trend differed between the two cell models, and the increased focus formation was not accompanied by anchorage–independent colony formation at 0.5 mg/mL. Consequently, the findings from these complementary assays did not provide clear evidence of malignant transformation under the tested conditions.

### 3.3 Genotoxicity Assessment of GY E500

#### 3.3.1 Ames Test

Samples expressing mutagenicity exceeding twice the frequency of spontaneous revertants and displaying a dose–response relationship were determined as positive (De Serres and Shelby 1979). The results of the Ames test with GY E500 are presented in Figure 5. The Ames test was conducted up to a maximum dose of 5000 μg/plate, and no cytotoxicity or mutagenic activity was observed in any tester strain, either with or without metabolic activation. No antibacterial effects were observed, as the colony counts were comparable to those of the control groups.

**FIGURE 5:**
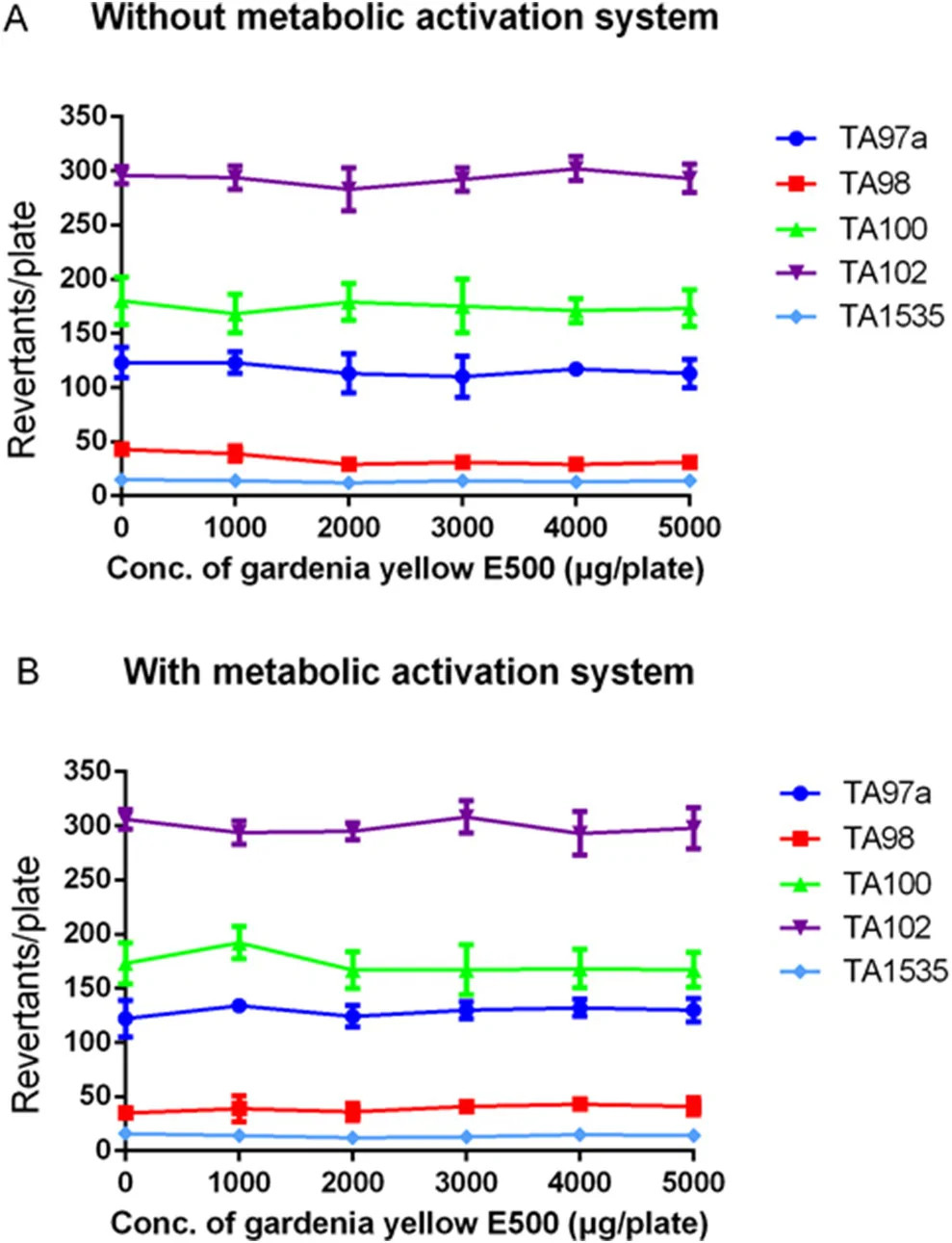
Effect of GY E500 on revertant colony formation in the Ames assay. (A) Without metabolic activation (−S9). (B) With metabolic activation (+S9). Five *S. typhimurium* tester strains (TA97a, TA98, TA100, TA102, TA1535) were exposed to GY E500 (0–5000 μg/plate). Data are mean ± SD (*n* = 3). Statistical analysis was performed by chi‐square test; *p* < 0.05 was considered significant.

As shown in Table 3, compared with the spontaneous regression control and positive control group, no significant increase (less than twice the spontaneous regression group) in the number of revertant colonies was observed for any dose or strain.

**TABLE 3:** The effect of GY E500 on revertant colonies in the Ames assay.

Group	S9	Bacteria strains				
		TA97a	TA98	TA100	TA102	TA1535
Solvent control	−	127 ± 15	41 ± 6	177 ± 17.5	289 ± 7	13 ± 2
	+	122.5 ± 18	33.5 ± 5.5	168.5 ± 16.5	300.5 ± 11	15 ± 3
Spontaneous regression	−	123.5 ± 18	35 ± 8.5	175 ± 16.5	291.5 ± 16	13 ± 2.5
	+	116.5 ± 15	33.5 ± 2	178.5 ± 19.5	297.5 ± 19.5	14.5 ± 3.5
Positive control	−	1112.5 ± 56^a^	928.5 ± 126.5^a^	1061.5 ± 82.5^b^	1192 ± 89^a^	634.5 ± 91.5^b^
	+	1175.5 ± 20.5^c^	908 ± 50.5^c^	1082 ± 129^c^	1110 ± 141^d^	993 ± 99.5^e^
GY E500 (128 μg/plate)	−	122 ± 11	38.5 ± 6	170 ± 23	294 ± 12	14 ± 3.5
	+	127.5 ± 9.5	39 ± 8	190 ± 16	300 ± 12.5	13 ± 2
GY E500 (320 μg/plate)	−	116 ± 12.5	35.5 ± 5.5	182 ± 17.5	292.5 ± 19.5	12.5 ± 3.5
	+	123.5 ± 7	36.5 ± 8.5	171.5 ± 12.5	293.5 ± 11.5	13 ± 2
GY E500 (800 μg/plate)	−	112.5 ± 10.5	36.5 ± 6	176.5 ± 24	296 ± 10	14 ± 4
	+	124 ± 7	36 ± 5.5	172 ± 20.5	300.5 ± 13	14.5 ± 3
GY E500 (2000 μg/plate)	−	116.5 ± 11.5	34.5 ± 4	173.5 ± 12	297.5 ± 11.5	13.5 ± 3.5
	+	127 ± 11	39 ± 6.5	172 ± 14	299.5 ± 13.5	14.5 ± 5
GY E500 (5000 μg/plate)	−	116.5 ± 9	35 ± 4	175.5 ± 14.5	298.5 ± 11.5	14 ± 2.5
	+	128 ± 13	38.5 ± 7	173.5 ± 13.5	298.5 ± 14	14.5 ± 4

^a^ Dexon, 50 μg per plate.

^b^ Sodium Azide, 1.5 μg per plate.

^c^ 2–Aminofluorene, 10 μg per plate.

^d^ 1,8–Dihydroxyanthraquinone, 50 μg per plate.

^e^ 2–Aminofluorene, 50 μg per plate.

#### 3.3.2 In Vitro Chromosomal Aberration Assay

The results of the in vitro chromosomal aberration assay are shown in Table 4. After 4–h treatment with GY E500 up to 5000 μg/mL, total chromosomal aberrations excluding gaps did not exceed 5.0% with or without the S9 mix. When gaps were included, total aberrations did not exceed 24.5% under either condition. These findings indicate that GY E500 was not clastogenic at the highest feasible concentration tested. As expected, the positive controls, cyclophosphamide (CP) and ethyl methanesulfonate (EMS), significantly increased aberrant metaphases. Laboratory–specific historical control data for the CHO–K1 chromosomal aberration assay are provided in Supplementary Table S2.

**TABLE 4:** The aberrant metaphases of CHO K1 cells in vitro chromosomal aberration assay for GY E500.

Group	Concentration (μg/mL)	S9	Aberrant metaphases excluding gaps and multiploid (%)	Aberrant metaphases including gaps and multiploid (%)
Negative Control	0	+	3	7
	0	−	1	10.5
GY E500	1250	+	2	9.5
	1250	−	0.5	8
	2500	+	1.5	10.5
	2500	−	1.5	13
	5000	+	5	24.5
	5000	−	2	23.5
Positive control				
Cyclophosphamide	15	+	12.5**	34
Ethyl Methanesulfonate	0.25	−	12.0**	35

**p < 0.01, significantly different from control.

#### 3.3.3 In Vivo Micronucleus Test

The incidence of micronucleated polychromatic erythrocytes (MNPCEs) per 2000 polychromatic erythrocytes (PCEs) is shown in Figure 6 and Table 5. Compared with the negative control, GY E500 did not significantly increase the MNPCE ratio in either female or male mice at doses of 1.665, 3.330, or 6.660 g/kg bw. In contrast, the positive control significantly increased the MNPCE ratio in both sexes (*p* < 0.01). Laboratory‐specific historical control data for MNPCE frequency and the PCE/(PCE + NCE) ratio are provided in Supplementary Table S3. No significant sex–related difference in MNPCE frequency was observed (*p* > 0.05). In addition, GY E500 did not markedly affect the PCE/(PCE + NCE) ratio, indicating no obvious bone marrow cytotoxicity under the tested conditions.

**FIGURE 6:**
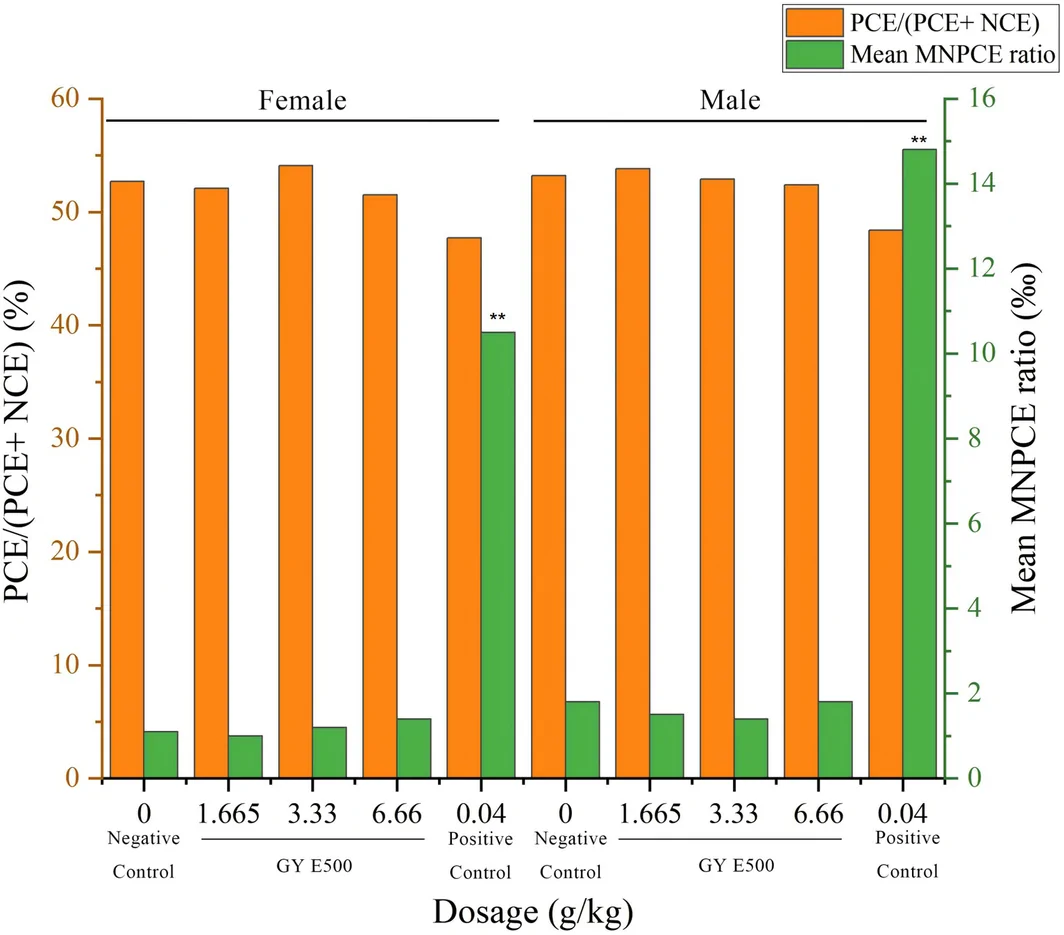
In vivo micronucleus assay of GY E500 in male and female mice, showing PCE/(PCE + NCE) (%) and MNPCE ratio (‰) after oral exposure (0, 1.665, 3.33, 6.66 g/kg bw). Cyclophosphamide (0.04 g/kg bw) served as the positive control. Data are mean ± SD (*n* = 5). Statistical analysis was performed by chi‐square test; **p < 0.01 only in the positive control.

**TABLE 5:** Comparison of in vivo micronucleus assay effects of different concentrations of GY E500 in mice.

Gender	Group	Dosage (g/kg bw)	Number of animals	PCE/(PCE+ NCE) (%)	MNPCE ratio (‰)
Female	Negative control	0.000	5	52.7	1.1 ± 0.7
	GY E500	1.665	5	52.1	1.0 ± 0.4
		3.330	5	54.1	1.2 ± 0.6
		6.660	5	51.5	1.4 ± 0.7
	Positive control (Cyclophosphamide)	0.04	5	47.7	10.5 ± 1.9**
Male	Negative control	0.000	5	53.2	1.8 ± 0.8
	GY E500	1.665	5	53.8	1.5 ± 0.5
		3.330	5	52.9	1.4 ± 0.7
		6.660	5	52.4	1.8 ± 0.4
	Positive control (Cyclophosphamide)	0.04	5	48.4	14.8 ± 2.8**

Abbreviations: NCE, mature red blood cells; PCE, polychromatic red blood cells.

**p < 0.01, significantly different from control.

Compared to the positive control, GY E500 at the doses of 1.66, 3.33, and 6.66 g/kg did not induce an increase in MNPCEs. The MNPCE frequencies, expressed as parts per thousand, found in the current study for the negative (♀:1.1 ± 0.7, ♂:1.8 ± 0.8) and positive controls (♀:10.5 ± 1.9, ♂:14.8 ± 2.8, *p* < 0.01) were within the expected range for Kunming mice (OECD 2016b). In addition, GY E500 at these doses did not significantly affect the PCE/(PCE + NCE) ratio, further indicating the absence of bone marrow cytotoxicity under the tested conditions.

## 4 Discussion

In the present study, GY E500, a commonly used high–color–value gardenia yellow product in the Chinese market, was selected as the test material to systematically evaluate its genotoxicity and carcinogenesis–related endpoints using an integrated strategy combining computational toxicology with in vitro and in vivo assays. GY is a natural food colorant widely used in East Asia. A recent review reported that China accounts for more than 80% of global gardenia yellow pigment production, with Chinese production projected to exceed 3000 t by 2025 (Yin et al. 2024). Chemically, GY is mainly composed of crocin, crocetin, and minor iridoid glycosides, including geniposide and its aglycone genipin (Ozaki et al. 2002), but systematic evidence regarding its genotoxicity and carcinogenicity remains limited and partly inconsistent. As a high–purity pigment product commonly used to prepare low–color–value GY products for direct food application, GY E500 may better reflect the toxicological properties of the pigment itself while reducing the potential influence of excipients (Tang et al. 2023). By integrating computational prediction with carcinogenesis‐related endpoints and standardized genotoxicity tests conducted according to the National Food Safety Standards for Food Safety Toxicological Evaluation Procedures (GB 15193–2014/2015), the present study did not identify consistent evidence of mutagenicity, chromosomal damage, micronucleus induction, or transformation–inducing activity under the tested conditions (Table S4). These findings supplement genotoxicity‐ and carcinogenicity–related safety evidence for GY E500 and may contribute to the evidence base supporting its future consideration for JECFA certification.

Initially, QSAR–based computational toxicology prediction was performed to characterize the ADMET properties and potential toxicity alerts of crocin and crocetin, the major constituents of GY. The predictions suggested that crocetin was mainly associated with non–genotoxic endpoints, including hepatotoxicity, eye irritation, and respiratory toxicity, without clear signals for genotoxicity or carcinogenicity, whereas one potential genotoxic carcinogenicity alert was identified for crocin in the structural alert analysis. Structurally and metabolically, crocin and crocetin are carotenoid–related compounds with conjugated polyene backbones; crocin is a glycosylated diester of crocetin that can be hydrolyzed to crocetin during gastrointestinal metabolism after oral exposure, whereas crocetin is a relatively hydrophobic diterpenoid polyene compound (Song et al. 2021). Although their structures are relatively complicated, neither compound contains aromatic amine, N–nitrosamine, or epoxide groups, which are recognized structural alerts for mutagenicity and genotoxic carcinogenicity (Benigni et al. 2008). This prediction pattern is consistent with previous studies showing that the chronic toxicity of GY under long–term or high–dose exposure conditions is mainly associated with hepatic and renal effects (Tang et al. 2020; Tian et al. 2018). However, QSAR predictions are influenced by training dataset coverage, model applicability domains, and the complexity of natural mixtures. For complex multi–component natural products such as GY, these models may have limited ability to fully capture the potential contributions of minor constituents, impurities, and metabolites. Therefore, the computational toxicology results provide preliminary clues for understanding the structural features and potential toxicity profile of the major constituents of GY E500.

The in vitro cell transformation assays further helped determine whether GY E500 could induce cellular transformation changes. In the transformation frequency assay, TF values were increased in the GY E500–treated groups compared with the negative control, although the concentration–related pattern differed between the two cell models. HaCaT cells showed a significant increasing trend among the GY E500–treated groups, whereas no significant trend was observed in SHEE cells. This discrepancy may be partly related to differences in cellular origin, as SHEE cells are derived from human esophageal epithelial cells and HaCaT cells from human keratinocytes, which may differ in their responsiveness to GY E500 or TPA–promoted transformation conditions. Therefore, although the increased TF values suggest that GY E500 exposure may influence transformation–related responses under the present experimental conditions, this finding should be interpreted cautiously because a consistent concentration–dependent pattern was not observed across both models. This inconsistency may also be associated with the low baseline frequency of transformation events, intrinsic differences in cellular sensitivity to TPA stimulation, and the limited non–cytotoxic concentration window (OECD 2007). Moreover, neither SHEE nor HaCaT cells formed colonies in soft agar, suggesting that GY E500 did not promote anchorage–independent growth, a key feature of malignant transformation. Taken together, these results do not support a transformation–inducing effect of GY E500 under the present assay conditions. Moreover, although direct evidence linking GY exposure to cancer risk remains limited, its components, such as crocin, crocetin, and genipin, have been reported to exhibit anticancer‐related activities, including inhibition of cancer cell proliferation, migration, and invasion (Koch et al. 2024; Natallia et al. 2024; Yang et al. 2023).

Our present study further examined the genotoxicity associated with GY. Although several studies have investigated this issue, the evidence remains inconsistent, particularly because most available data were derived from in vitro assays, whereas in vivo evidence remains limited. Ozaki et al. reported that GY and genipin induced DNA damage in the rec–assay and significantly increased SCE frequency, leading to the conclusion that genipin rather than GY as a whole was the main driver of genotoxicity (Ozaki et al. 2002). In a contrasting study, although genipin yielded positive results in an in vitro micronucleus and chromosome aberration assay under several test conditions, it displayed equivocal results in vivo tests conducted in mice (Hobbs et al. 2018). Moreover, an in vitro study utilizing CHO–K1 cells by Tsai et al. found that genipin did not cause genotoxicity at concentrations below 50 ppm (Tsai et al. 2000). Regarding crocin and its derivatives, the main components of GY, Hosseinzadeh et al. found that crocin did not exhibit genotoxic effects and instead produced a dose–dependent reduction in DNA damage, as evidenced by the comet assay (Hosseinzadeh et al. 2008). Additionally, Pawar et al. reported that crocin showed no genotoxicity in the Ames assay across a dosage range of 125 to 2000 μg (Pawar et al. 2019). Taken together, these findings suggest that the genotoxic potential of GY may largely depend on the relative contribution of specific components, particularly genipin, and on the test system employed.

Based on OECD guidance, this study evaluated the genotoxicity of GY using multiple genotoxicity assays covering different endpoints. Consistent with most of the existing literature, negative results were obtained in the Ames test and the micronucleus test. Results of the in vitro chromosomal aberration assay showed that the frequency of true structural aberrations for GY E500 at concentrations up to 5000 μg/mL did not exceed 5.0% with or without S9 activation, although the total frequency reached 24.5% when gaps were included. Since OECD TG 473 does not generally consider chromatid and chromosome gaps to be biologically relevant, the observed increases, including gaps, were not interpreted as evidence of clastogenicity, and the incidence of true structural aberrations remained low and within background variation (OECD 2016a). The tested GY E500 sample contained 44.82% (w/w) crocin, crocetin, and their derivatives, whereas geniposide and genipin accounted for 0.214% and 0.03% (w/w), respectively (Tang et al. 2020). Accordingly, even at the highest tested concentrations in our genotoxicity assays, the actual exposure level of genipin remained below the 50 ppm concentration at which Tsai et al. reported no genotoxic effects. Together with the negative Ames and micronucleus results and the absence of biologically relevant increases in true structural chromosomal aberrations, these findings indicate that GY E500 did not exhibit genotoxic potential under the tested conditions.

This study, through integrated in silico and in vitro carcinogenesis–related screening assays and standardized genotoxicity analyses, found no evidence of genotoxicity or transformation–inducing activity of GY E500, and no findings indicative of carcinogenesis–related concern, providing new evidence for the GY's risk assessment. Similar integrated approaches combining in silico prediction with experimental validation have increasingly been applied in recent natural product research (Kiran et al. 2025). Consistent with these developments, this step–wise multi–level framework enables early risk prediction and stage–wise experimental validation by integrating complementary evidence from computational, in vitro, and in vivo approaches, thereby reducing reliance on individual assays and potentially limiting unnecessary animal use, while providing a more reliable evaluation of GY genotoxicity and cell transformation potential and a methodological reference for applying NAMs to food additive safety assessment. However, there still exist some uncertainties in the present study. Firstly, computational predictions may be affected by the limited coverage of available prediction databases and model applicability domains, which may result in potential false–positive or false–negative predictions. Epithelial–derived cell lines provide a useful experimental window into initial toxicological events at mucosal barriers; however, their epithelial origin and limited endpoints may introduce uncertainties in extrapolating to the in vivo context. Future studies incorporating liver–derived models and detailed analyses of molecular damage mechanisms would improve the accuracy of extrapolation and further strengthen the safety assessment. Furthermore, long–term in vivo animal studies are needed to clarify potential effects under chronic exposure, and the inherent limitations of such data, together with interspecies toxicokinetic and toxicodynamic differences, should be considered when extrapolating these findings to humans. Moreover, variations in crocin, crocetin, and geniposide levels may occur due to differences in producing areas or extraction methods, the batch–to–batch consistency of GY E500 requires further evaluation. However, the present study evaluated a high–chromatic–value GY sample produced using standardized protocols, which represents a typical product used in the Chinese market. In addition, the concentrations used in in vitro assays (0.1–10 mg/mL) and the oral doses applied in the in vivo micronucleus test (up to 6.66 g/kg bw) were significantly higher than estimated human dietary exposure levels. Such high–dose ranges are commonly employed in toxicological testing to maximize sensitivity and to provide a conservative assessment of potential hazards. Nevertheless, long–term repeated dietary exposure and inter–individual variability in metabolism may influence internal exposure to GY constituents and should be considered in future exposure and toxicokinetic evaluations. In this study, the upper in vivo dose was determined by the solubility of GY E500 and the gavage volume limit, which is consistent with OECD Test Guideline 474. Although these doses exceed realistic intake scenarios by several orders of magnitude, the absence of genotoxic effects at such high doses supports a wide margin of safety for GY when used within regulatory specifications, including the ≤ 1% limit for geniposide (Tang et al. 2020; JECFA 2015).

## 5 Conclusions

In summary, the integrated in silico, in vitro and in vivo assessment of a representative GY E500 product from the Chinese market did not indicate genotoxicity or provide evidence suggestive of cell transformation under the tested experimental conditions. These findings provide additional evidence for the genotoxicity– and cell transformation–related safety profile of GY and support its consideration as a natural food colorant. Nevertheless, given the intrinsic complexity of natural products and the absence of long–term in vivo data, further studies focusing on chronic exposure scenarios are warranted to strengthen the overall safety evaluation.

## 6 Limitations of the Study

1. Computational predictions are influenced by the coverage and applicability domain of available databases.
2. The assessment of transformation–inducing potential was mainly based on in vitro evidence, with limited long–term data and uncertainty in extrapolation from cell models to mice.

## Author Contributions

**Jiang Liang:** methodology, writing – review and editing, supervision, funding acquisition. **Min Qu:** investigation, data curation, software. **Yingying Zhou:** software, writing – original draft, writing – review and editing. **Xiaoqiao Tang:** investigation. **Yuan Yuan:** investigation. **Yinjing Zhang:** methodology. **Haibin Xu:** project administration. **Wei Liu:** data curation. **Haitao Han:** investigation. **Lei Zhang:** conceptualization, resources, funding acquisition. **Jintao Li:** conceptualization, resources, supervision, project administration.

## Funding

The study was supported by the China Food Safety Talent Competency Development Initiative (CFSA 523 Program) and Guangdong Key R&D Program (No. 2019B020210002).

## Conflicts of Interest

The authors declare no conflicts of interest.

## Supporting information


**Table S1:** Colony‐Forming Unit Assays and Malignant transformation of SHEE cells and HaCaT cells induced by GY E500.
**Table S2:** Historical Data for the In Vitro Chromosomal Aberration Assay in CHO‐K1 Cells.
**Table S3:** Historical Data for the Bone Marrow Micronucleus Assay in Kunming Mice.
**Table S4:** Summary of the step‐wise integrated assessment of GY E500.

## Data Availability

The data that support the findings of this study are available from the corresponding author upon reasonable request.
