# Supplementary material for: In Silico, In Vitro, and In Vivo Assessment of Genotoxicity and Cell Transformation Potential of Gardenia Yellow E500
Source: Food Sci Nutr. 2026 Nov 13;14(11):e72552. doi: 10.1002/fsn3.72552 (PMC13768739; doi:10.1002/fsn3.72552)
Supplement: Supplementary file 1 [file FSN3-14-e72552-s001.docx]

Table S1 Colony-Forming Unit Assays and Malignant transformation of SHEE cells and HaCaT cells induced by GY E500

| Cell line | Concentration  (mg/mL) | Number of Clones | CE | Number of transformation *foci* | TF  (×10^-4^, Mean ± SD) |
| --- | --- | --- | --- | --- | --- |
| SHEE cells | 0 | 245 | 0.612 | 0 | 0 |
|  | 0.1 | 212 | 0.53 | 1 | 1.70 ± 0.06 |
|  | 0.5 | 200 | 0.5 | 1 | 1.85 ± 0.05 |
|  | 1 | 185 | 0.462 | 2 | 1.75 ± 0.06 |
| HaCaT cells | 0 | 236 | 0.59 | 1 | 1.30 ± 0.06 |
|  | 0.1 | 207 | 0.518 | 2 | 1.55 ± 0.04 |
|  | 0.5 | 181 | 0.453 | 2 | 1.93 ± 0.05 |
|  | 1 | 175 | 0.438 | 5 | 2.34 ± 0.07 |

Note:

1. The experiment was performed in triplicate, and the data are presented as the mean of three replicate trials.

2. The malignant transformation experiments were conducted using a culture medium containing 0.1 μg/mL TPA in DMEM/F12 supplemented with 2% FBS, 0.5% P.S., 0.5% Glu (Glucose), and 0.2% ITES

Table S2 Historical Data for the *In Vitro* Chromosomal Aberration Assay in CHO-K1 Cells

| Control group | Exposure duration | S9 activation | n | Historical mean ± SD (%) | Historical range (%) | Concurrent control (mean, %) |
| --- | --- | --- | --- | --- | --- | --- |
| Negative control | 4 h | + | 28 | 3.1 ± 0.8 | 1.5-4.7 | 3.0 |
| Negative control | 4 h | − | 28 | 1.7 ± 0.8 | 0.9-2.5 | 1.0 |
| Cyclophosphamide (1.5 mg/mL) | 4 h | + | 28 | 20.2 ± 4.0 | 12.3-28.1 | 12.5 |

Table S3 Historical Data for the Bone Marrow Micronucleus Assay in Kunming Mice

| Endpoint | Control group | Sex | sample size | Range | Concurrent control  (mean) |
| --- | --- | --- | --- | --- | --- |
| MNPCE frequency (‰) | Negative control | Male | 90 | 0–5.4 | 1.8 |
|  |  | Female | 70 | 0–5.6 | 1.1 |
|  | CP, 0.04 g/kg bw | Male | 90 | 8.2–30.1 | 14.8 |
|  |  | Female | 70 | 7.6–25.1 | 10.5 |
| PCE/(PCE + NCE)  (%) | Negative control | Male | 90 | 47.9–63.0 | 53.2 |
|  |  | Female | 70 | 47.7–59.9 | 52.7 |
|  | CP, 0.04 g/kg bw | Male | 90 | 42.6–55.4 | 48.4 |
|  |  | Female | 70 | 44.4–55.4 | 47.7 |

Table S4 Summary of the step-wise integrated assessment of GY E500

| Assessment level | Assay | Endpoint | Overall result | Interpretation |
| --- | --- | --- | --- | --- |
| *In silico* | ADMETlab 2.0 and admetSAR 2.0 | Genotoxicity and carcinogenicity prediction | One potential structural alert for crocin | No consistent genotoxicity or carcinogenicity signal |
| *In vitro* | Colony-forming and malignant transformation assays | Transformation-  related responses | No consistent positive response | No consistent transformation-inducing effect under the tested conditions |
| *In vitro* | Soft agar colony formation assay | Anchorage-  independent growth | Negative | No evidence of anchorage-independent growth |
| *In vitro* | Ames test | Gene mutation | Negative | No evidence of mutagenicity |
| *In vitro* | Chromosomal aberration assay | Structural chromosomal damage | Negative | No biologically relevant increase in true structural chromosomal aberrations |
| *In vivo* | Micronucleus assay | Micronucleus induction | Negative | No evidence of micronucleus induction in vivo |

## Supplementary Methods: In silico toxicological prediction

## ADMETlab 2.0 prediction

ADMETlab 2.0 was used to predict the ADMET and toxicity properties of crocin and crocetin, the two major constituents of GY. ADMETlab 2.0 was selected because it provides systematic evaluation of multiple ADMET and toxicity endpoints based on curated experimental datasets and validated QSAR models. The applicability and reliability of the predictions were considered according to the endpoint scope, curated training datasets, physicochemical descriptor space, platform output definitions, and model validation information reported in the original ADMETlab 2.0 publication. The SMILES strings of crocin and crocetin were obtained from PubChem and used as structural inputs for prediction. In this study, 27 toxicity endpoints were evaluated, including 11 animal toxicity endpoints, 4 environmental toxicity endpoints, and 12 toxicological pathway endpoints. In addition, 8 toxicophore alert categories were assessed. The prediction results were recorded according to endpoint categories, including animal toxicity, environmental toxicity, toxicological pathways, and toxicophore alerts. For categorical endpoints, ADMETlab 2.0 provides both qualitative symbols and numerical output values. The numerical output value represents the predicted probability of the corresponding toxicity or activity. According to the platform output format, “---”, “--”, “-”, “+”, “++”, and “+++” indicate increasing probability levels from low to high. The interpretation of the prediction results was based on the output definitions of ADMETlab 2.0 and the model validation information reported in the original platform publication.

## admetSAR 2.0 prediction

admetSAR 2.0 was used as another QSAR-based prediction platform to compare with the results obtained from ADMETlab 2.0. The same SMILES strings of crocin and crocetin obtained from PubChem were submitted to admetSAR 2.0, ensuring consistent structural inputs across the two platforms. admetSAR 2.0 was selected because it integrates a large ADMET knowledgebase and machine-learning models for multiple toxicity endpoints. According to the original admetSAR 2.0 publication, model applicability was considered based on an applicability-domain definition using physicochemical and topological properties, including molecular weight, AlogP, numbers of hydrogen-bond donors and acceptors, and numbers of atoms and rings. In this study, 18 toxicity endpoints were evaluated, including 7 animal toxicity endpoints, 6 toxicological pathway endpoints, and 5 ecotoxicity endpoints. The outputs from admetSAR 2.0 were recorded as qualitative prediction results and “possibility” values. In the platform output, “+” indicates a positive or toxic prediction, whereas “−” indicates a negative or non-toxic prediction. The “possibility” value represents the platform-generated probability of the predicted class and was used as the confidence output. The interpretation of the prediction results was based on the output definitions of admetSAR 2.0 and the model validation information reported in the original platform publications.
